# Supplementary material for: Post-Harvest Contamination with Mycotoxins in the Context of the Geographic and Agroclimatic Conditions in Romania
Source: Toxins (Basel). 2018 Dec 13;10(12):533. doi: 10.3390/toxins10120533 (PMC6316900; doi:10.3390/toxins10120533)
Supplement: Supplementary file 1 [file toxins-10-00533-s001.pdf]

Supplementary Materials: Post-Harvest Contamination with Mycotoxins in the Context of the Geographic and Agroclimatic Conditions in Romania

Valeria Gagiu, Elena Mateescu, Ileana Armeanu, Alina Alexandra Dobre, Irina Smeu, Mirela Elena Cucu, Oana Alexandra Oprea, Enuta Iorga and Nastasia Belc

Table S1. Differences in the average air temperature by region and year in Romania in 2012–2015 (ANOVA two-factors without replications).

**Differences in the Average Air Temperature by Region and Year in Romania (March–August, 2012–2015)**						
**Source of Variation**	**SS**	**df**	**MS**	***F***	***p*-value**	***F* crit.**
**Region**	23.10	5	4.62	101.49	0.000	2.90
**Year**	5.76	3	1.92	42.21	0.000	3.29
**Error**	0.68	15	0.05			
**Total**	29.55	23				

SS—sum of the squares; df—degrees of freedom; MS—the mean sum of squares; *F*—Fisher test;.*p*-value—probability value; *F* crit.—the critical value of *F* test.

Table S2. Class divisions of the average air temperature by region in Romania in 2012—2015 (*t*-test multiple).

**Class Division**	**Agricultural Region**	**Average Air Temperature ± SD, °C**	**Differences in the Average Air Temperature by Region in Romania (March–August, 2012–2015)**				
			**Dif. 1**	**Dif. 2**	**Dif. 3**	**Dif. 4**	**Dif. 5**
I	**Oltenia Plain**	18.0 ± 0.80	2.93 ***	1.75 ***	1.20 ***	0.95 ***	0.20 ns
	**Southern Plain and Dobrogea**	17.8 ± 0.62	2.73 ***	1.55 ***	1.00 ***	0.75 ***	0
II	**Southern Hilly Area**	17.1 ± 0.58	1.98 ***	0.80 ***	0.25 ns	0	
	**Western Plain**	16.8 ± 0.45	1.73 ***	0.55 ***	0		
III	**Moldavia**	16.3 ± 0.65	1.18 ***	0			
IV	**Transylvania**	15.1 ± 0.41	0				
DL 5% = 0.29, DL 1% = 0.40, DL 0.1% = 0.54							

Dif.—difference; ns—non-significant difference; ***—difference is significant at the 0.001 level (two-tailed); DL—difference limit.

Table S3. Class division of the average air temperature by year in Romania, 2012–2015 (*t*-test multiple).

Class Division	Agricultural Year	Average Air Temperature ± SD, °C	Differences in the Average Air Temperature by Year in Romania (March–August, 2012–2015)		
			Dif. 1	Dif. 2	Dif. 3
I	2012	17.62 ± 1.56	1.30 ***	1.07 ***	0.80 ***
II	2015	16.83 ± 0.90	0.50 **	0.27 *	0
III	2013	16.55 ± 1.36	0.23 ns	0	
	2014	16.32 ± 0.93	0		
DL 5% = 0.26, DL 1% = 0.36, DL 0.1% = 0.50					

Dif.—difference; ns—non-significant difference; *—difference is significant at the 0.05 level (two-tailed); **—difference is significant at the 0.01 level (two-tailed); ***—difference is significant at the 0.001 level (two-tailed). DL—difference limit.

Table S4. Differences in the average cumulative precipitation by region and year in Romania in 2012–2015 (ANOVA two-factors without replications).

Differences in the Average Cumulative Precipitation by Region and Year in Romania (March–August, 2012–2015)						
Source of Variation	SS	df	MS	*F*	*p*-value	*F* crit.
Region	33664.00	5	6732.80	1.80	0.172	2.90
Year	134190.33	3	44730.11	11.99	0.000	3.29
Error	55951.65	15	3730.11			
Total	223806.08	23				

SS—sum of the squares; df—degrees of freedom; MS—the mean sum of squares; *F*—Fisher test; *p*-value—probability value; *F* crit.—the critical value of *F* test.

Table S5. Class divisions of the average cumulative precipitation by year in Romania in 2012–2015 (t-test multiple).

Class Division	Agricultural Year	Average Cumulative Precipitation ± SD, mm	Differences in the Average Cumulative Precipitation by Year in Romania (March–August, 2012–2015)		
			Dif. 1	Dif. 2	Dif. 3
I	2014	474.43 ± 99.82	180.57 ***	172.86 ***	96.00 *
II	2013	381.14 ± 48.87	87.28 **	79.57 *	0
III	2012	301.57 ± 41.63	7.71 ns	0	
	2015	293.86 ± 31.73	0		
DL 5% = 63.35, DL 1% = 86.17, DL 0.1% = 117.76					

Dif.—difference; ns—non-significant difference; *—difference is significant at the 0.05 level (two-tailed); **—difference is significant at the 0.01 level (two-tailed); ***—difference is significant at the 0.001 level (two-tailed); DL—difference limit.

Table S6. Comparison of the average values of agrometeorological factors between agricultural regions and years in Romania in 2012–2015 (Kruskal–Wallis non-parametric test for independent samples, when the null hypothesis was rejected).

No.	**Null Hypothesis**	***N***	**Significance Level**
1	The distribution of average air temperature is the same across categories of agricultural region	153	0.000
2	The distribution of cumulative precipitation is the same across categories of agricultural region	153	0.018
3	The distribution of soil moisture reserve is the same across categories of agricultural region	152	0.000
4	The distribution of soil moisture reserve is the same across categories of year	152	0.012
